# Students’ Attitude Change: Virtual vs. In-Person Intergenerational, Arts-Based Courses

**DOI:** 10.1093/geroni/igab046.1414

**Published:** 2021-12-17

**Authors:** Meghan Young, Elizabeth Lokon, Yue Li

**Affiliations:** 1 Scripps Gerontology Center, Miami University, Oxford, Ohio, United States; 2 Miami University, Oxford, Ohio, United States

## Abstract

When higher education classes went virtual at the start of the COVID-19 pandemic, converting an in-person, arts-based, service-learning course into a meaningful, virtual experience seemed impossible. However, the Opening Minds through Art (OMA) program developed online courses where students met older adults weekly over Zoom to create and discuss art. Undergraduate and graduate students at Miami and Marian Universities (n=47) came from more than 20 different areas of study and had varying knowledge of gerontology and dementia. Pre- and post-assessments were administered at the start and end of the academic semester. Paired-samples t-tests were conducted to examine pre-post changes in students’ attitudes toward people living with dementia (PLWD) using the Dementia Attitude Scale (DAS) (O’Connor & McFadden, 2010) and the extent students actually like PLWD using the Allophilia scale (Pittinsky et al, 2011). Students in virtual OMA courses showed significant improvement in overall DAS and Allophilia scores and all subdomain scores (i.e., general knowledge about dementia, affection, social comfort level, kinship, and engagement and enthusiasm when interacting with PLWD), with moderate to high effect sizes (Cohen’s d range between 0.39 and 1.10). The magnitudes of these effect sizes for virtual OMA are comparable to previous studies examining students’ participation in face-to-face OMA sessions, where Cohen’s d on DAS and Allophilia scales ranged between 0.48 and 1.07 (Lokon et al, 2017, 2018). Overall, we found that it is possible to design virtual service-learning courses that improve students’ attitudes toward PLWD as effectively as face-to-face courses.

